# Biomimetic Response of Zr-2.5Nb Alloy to Artificial Saliva with Variable pH: Corrosion Behavior and Surface Adaptation

**DOI:** 10.3390/biomimetics11030176

**Published:** 2026-03-02

**Authors:** Viorica Ghisman, Nicoleta Bogatu, Elena Emanuela Herbei, Alina Crina Muresan, Daniela Laura Buruiana

**Affiliations:** Interdisciplinary Research Centre in the Field of Eco-Nano Technology and Advance Materials CC-ITI, Faculty of Engineering, “Dunarea de Jos” University of Galati, 47 Domneasca, 800008 Galati, Romania; viorica.ghisman@ugal.ro (V.G.); nicoleta.simionescu@ugal.ro (N.B.); elena.herbei@ugal.ro (E.E.H.); alina.muresan@ugal.ro (A.C.M.)

**Keywords:** Zr–2.5Nb alloy, biomimetics, dental biomaterials, artificial saliva, oral corrosion, pH-dependent degradation

## Abstract

The long-term performance of metallic biomaterials in the oral environment is strongly influenced by their interaction with saliva and its variable chemical conditions. In this study, the biomimetic behavior of a Zr-2.5Nb alloy was investigated during immersion in artificial saliva with acidic, neutral, and alkaline pH for a period of 28 days, aiming to simulate diverse physiological and pathological oral conditions. The evolution of saliva pH was continuously monitored throughout the immersion period to assess the dynamic material–environment interactions. Surface morphology, elemental composition, and chemical structure were analyzed using scanning electron microscopy coupled with energy-dispersive X-ray spectroscopy (SEM/EDX) and Fourier-transform infrared spectroscopy (FTIR), while corrosion resistance was evaluated through electrochemical measurements. The results revealed distinct pH-dependent surface responses, with acidic saliva promoting localized surface modifications and increased corrosion susceptibility, whereas neutral conditions favored the formation of stable and protective passive layers, and alkaline environments promoted the development of chemically complex surface layers that exhibited initial stabilization but underwent progressive degradation during long-term exposure. Overall, the Zr-2.5Nb alloy demonstrated a high degree of corrosion resistance and chemical stability, particularly under neutral and alkaline conditions, supporting its suitability for dental and oral biomedical applications. These findings provide a biomimetic perspective on pH-driven surface adaptation and long-term corrosion behavior, highlighting how dynamic material–environment interactions govern the performance of zirconium-based biomaterials in complex oral environments.

## 1. Introduction

Zirconium-based alloys have recently emerged as promising candidates for biomedical implants, primarily due to their capacity to form bone-like apatite layers under physiological conditions and their intrinsically low magnetic susceptibility, which supports compatibility with magnetic resonance imaging techniques [1,2,3]. Commercially pure zirconium (CP Zr) and zirconium-based alloys exhibit a favourable combination of physical and mechanical properties, including low density (~6.5 g·cm^−3^), tensile strength in the range of 440 MPa for CP Zr and ~240 MPa for CP Ti, Young’s modulus between ~88 and 103 GPa, and very low magnetic susceptibility (~1.3–3.2 × 10^−6^ cm^3^·g^−1^), making them suitable for applications requiring mechanical reliability and magnetic compatibility [4]. Zirconium alloys are commonly classified into industrial and nuclear grades based on their alloying elements and impurity limits, with Zr–Nb alloys such as Zr–2.5Nb belonging to the industrial alloy category, characterized by controlled Nb contents (~2–3 wt%), low oxygen levels (<0.18 wt%), and enhanced mechanical strength and corrosion resistance compared to CP Zr, while maintaining low magnetic susceptibility and structural stability [5,6]. The incorporation of niobium into zirconium has been shown to enhance both mechanical strength and corrosion resistance [7,8]. Owing to its balanced combination of mechanical performance, corrosion stability, and biocompatibility, wrought Zr–2.5Nb alloy has been standardized for orthopedic implant applications [9]. Beyond the wrought and annealed condition, as-cast Zr–Nb alloys with different niobium contents have attracted attention due to their favorable mechanical behavior and low magnetic susceptibility, supporting their use in medical devices compatible with magnetic resonance imaging [7]. A comparative study on Zr–2.5X (X = Nb, Sn) alloys for biomedical applications reported that both Nb and Sn alloying additions contribute to strengthening zirconium and improving its corrosion behavior; however, the presence of Nb favors the formation of a duplex α+β microstructure, enhances resistance to localized corrosion, and preserves low magnetic susceptibility, while maintaining good in vitro cytocompatibility in physiological environments [10]. Previous studies have also reported that cold-rolled Zr–Nb alloys exhibit promising MRI compatibility, further broadening their potential biomedical applications. In addition to biocompatibility and magnetic compatibility, zirconium-based alloys can undergo surface thermal oxidation at elevated temperatures, leading to the formation of hard, dense, and adherent ceramic oxide layers with enhanced wear resistance [11]. Consequently, surface-oxidized Zr–2.5Nb alloys have been successfully applied in artificial knee and hip joints, while oxidized Zr–Sn alloys have been identified as promising candidates for oral implantology applications [12,13,14]. Previous investigations on Zr–Nb alloys have shown that grain refinement and phase homogenization achieved by severe plastic deformation (ECAP) can reduce the grain size to the sub-200 nm range and decrease the corrosion current density by up to ~88% for Zr–1 wt.% Nb and ~28% for Zr–2.5 wt.% Nb, while significantly altering passivation and pitting behavior in physiological solutions as a function of Nb content and microstructural state [15]. Recent investigations on bidirectionally hot-forged Zr–Nb alloys have shown that controlled thermomechanical processing within 800–900 °C and low strain rates (0.001–0.01 s^−1^) promotes dynamic recrystallization and significant α-phase refinement, leading to direction-dependent hardness increases of up to ~35%, which is highly relevant for tailoring the mechanical reliability and microstructural stability of zirconium-based biomaterials intended for load-bearing orthopedic and dental applications [16]. Previous electrochemical investigations of Zr–2.5Nb alloy in Ringer’s solution reported rapid surface stabilization within the first 20 h of immersion, followed by a quasi-equilibrium state with a slight shift toward more positive potentials, while polarization resistance and cyclic voltammetry analyses indicated a limited decrease in corrosion resistance over time and a susceptibility to localized pitting corrosion, subsequently confirmed by optical microscopy [17]. Recent investigations have demonstrated that variations in the pH and chemical composition of artificial saliva significantly influence the corrosion behavior and surface integrity of dental metallic materials, with acidic conditions promoting pitting and selective metal leaching, while neutral and alkaline environments can stabilize or modify the passive layer, thereby affecting long-term biocompatibility and mechanical performance in the oral environment [18].

Recent corrosion studies on Zr–Nb alloys in simulated physiological and salivary environments have predominantly focused on short-term exposure, static pH conditions, or isolated electrochemical parameters. Although these investigations provide valuable insight into passive film formation and general corrosion resistance, they often overlook long-term surface evolution, dynamic pH changes, and the distinction between chemically complex surface layers and truly protective passive films. Moreover, alkaline conditions frequently encountered in oral environments due to dietary habits or pathological states remain insufficiently explored, particularly in terms of their long-term electrochemical implications [19,20,21].

Despite extensive research on the corrosion behavior of zirconium-based alloys in physiological solutions and artificial saliva, most previous studies have focused on short-term exposure, fixed pH conditions, or single characterization techniques. In particular, the combined effects of long-term immersion, dynamic pH evolution, and correlated surface–electrochemical responses, especially under alkaline conditions, remain insufficiently explored. Moreover, the distinction between true passive film formation and porous surface deposition layers has often been overlooked.

In this context, the present study addresses these gaps by providing a biomimetic, time-resolved assessment of a Zr–2.5Nb alloy in artificial saliva with acidic, neutral, and alkaline pH, systematically correlating pH evolution with surface morphology (SEM–EDX), surface chemistry (FTIR), and electrochemical stability. This integrated dataset enables a more nuanced understanding of pH-driven surface adaptation versus long-term corrosion degradation, offering new insights relevant to dental and oral biomedical applications.

## 2. Materials and Methods

### 2.1. Materials Preparation

Zr–2.5Nb alloy plates with dimensions of 25 mm × 25 mm × 2 mm were used in this study. Prior to immersion tests, all samples were mechanically polished to obtain a smooth and uniform surface representative of clinical conditions. The polishing procedure was followed by ultrasonic cleaning in ethanol and distilled water to remove surface contaminants and residues. After cleaning, the samples were dried in air and stored in a desiccator until further use.

### 2.2. Preparation of Artificial Saliva

Artificial saliva was employed to simulate the physicochemical and biochemical conditions of the human oral environment, enabling the assessment of the alloy’s behavior under conditions relevant to dental and oral biomedical applications. The use of artificial saliva ensures experimental reproducibility and allows strict control of chemical composition and pH, which are critical parameters influencing corrosion and surface interactions. The artificial saliva solution was prepared according to the Fletcher method [18]. The base solution consisted of 0.2 mM MgCl_2_, 1 mM CaCl_2_, 4 mM Na_2_HPO_4_, 16 mM KCl, and 4.5 mM NH_4_Cl, dissolved in a 20 mM HEPES buffer. HEPES buffer (≥99% purity) was used to stabilize the solution pH. Distilled water was used for all solution preparations. Prior to use, 300 ppm sodium fluoride (NaF) was added to all solutions. The addition of 300 ppm sodium fluoride (NaF) was selected to reflect fluoride concentrations commonly encountered in the oral environment because of routine exposure to fluoridated toothpaste, mouth rinses, and professional dental prophylactic treatments. This fluoride level is widely used in artificial saliva formulations and is considered clinically relevant for evaluating corrosion and surface interactions of dental biomaterials.

After preparation, the Zr–2.5Nb samples were immersed in artificial saliva with acidic, neutral, and alkaline pH for a period of 28 days, after which they were removed and analyzed by SEM–EDX and FTIR to investigate surface morphology and chemical evolution.

Electrochemical corrosion measurements were performed on separate sets of samples and extended up to 2 months, with testing conducted at 0 days, 7 days, 1 month, and 2 months, in order to evaluate the time-dependent corrosion behavior and long-term stability of the passive surface layers.

### 2.3. pH Adjustment of Artificial Saliva

Three types of artificial saliva with different pH values were prepared to simulate acidic, neutral, and alkaline oral environments. For all solutions, the total volume of artificial saliva was 200 mL, corresponding to the electrolyte volume used during immersion and electrochemical testing.

S1—Acidic artificial saliva:

The base artificial saliva solution was adjusted by adding 0.2 g of uric acid to 200 mL of solution. The pH was experimentally measured after preparation and was 6.83 after 1 day, gradually increasing during immersion, reaching 8.16 after 28 days.

S2—Neutral artificial saliva:

The base artificial saliva solution was used without additional pH modifiers. The initial pH was 7.75, remaining close to physiological values throughout the immersion period (pH ≈ 7.31–7.40).

S3—Alkaline artificial saliva:

The base artificial saliva solution was adjusted by adding 0.2 mL of NaOH aqueous solution (analytical grade) to 200 mL of artificial saliva. The initial pH measured after preparation was 8.22, followed by a decrease and stabilization around pH 7.40 during immersion.

The pH of each solution was measured using a calibrated pH meter prior to immersion and periodically monitored throughout the experimental period to evaluate the dynamic interaction between the alloy surface and the surrounding medium.

### 2.4. Immersion Tests

Following preparation, the Zr–2.5Nb samples were fully immersed in the three types of artificial saliva (S1, S2, and S3) and maintained under controlled laboratory conditions for a period of 28 days. During immersion, the evolution of the saliva pH was periodically monitored to evaluate the dynamic interaction between the alloy surface and the simulated oral environment.

Two distinct experimental protocols were employed. Samples used for surface characterization (SEM–EDX and FTIR) were immersed in artificial saliva for 28 days. Electrochemical corrosion measurements were performed on separate sets of specimens, which were exposed to artificial saliva for up to 2 months, with electrochemical testing conducted at 0 days, 7 days, 1 month, and 2 months. For electrochemical experiments, independent specimens were used for each exposure time, and samples were not reintroduced into the solution after testing. During immersion and electrochemical testing, the artificial saliva solution was not renewed, in order to allow natural pH evolution and solution surface interactions, consistent with the biomimetic objective of the study. For electrochemical measurements, the samples were masked to expose a defined working area of 5.06 cm^2^, while the remaining surface was electrically insulated to avoid edge effects and ensure accurate current density calculations. All immersion and electrochemical experiments were conducted at room temperature (23 ± 2 °C) under controlled laboratory conditions.

### 2.5. Characterization Methods

The surface morphology and elemental composition of the Zr–2.5Nb alloy samples were investigated using a Tescan Vega scanning electron microscope equipped with an energy-dispersive X-ray spectroscopy (EDX) detector. SEM analysis was employed to assess surface topography and morphological changes induced by immersion in artificial saliva with different pH values, including surface irregularities, localized corrosion features, and surface deposits. EDX analysis provided qualitative and semi-quantitative information on the elemental distribution at the sample surfaces, enabling the identification of zirconium, niobium, oxygen, and carbon, as well as potential compositional changes associated with surface oxidation and interactions with the simulated oral environment. The large specimen chamber facilitated efficient handling and analysis of multiple samples under stable vacuum conditions.

FTIR analysis was performed using a Shimadzu spectrophotometer equipped with a QATR-S attenuated total reflectance (ATR) accessory incorporating a diamond crystal. Spectra were recorded wavenumber range of 4000–400 cm^−1^, to identify chemical functional groups present on the surface of the Zr–2.5Nb alloy after immersion in artificial saliva. FTIR spectroscopy was used to investigate pH-dependent chemical changes and the formation of oxide, hydroxyl, carbonate, and organic-related functional groups resulting from interactions between the alloy surface and the simulated oral environment.

The electrochemical corrosion behavior of the Zr–2.5Nb alloy samples was investigated using a multichannel potentiostat/galvanostat (OrigaLys, France; OrigaFlex model OGF+01A) controlled by OrigaMaster OM 5 software. Measurements were carried out in artificial saliva under controlled laboratory conditions at four exposure intervals: initial condition 0 days, 7 days, 1 month, and 2 months. A conventional three-electrode electrochemical cell was used, consisting of the Zr–2.5Nb alloy sample as the working electrode, an Ag/AgCl reference electrode with saturated KCl solution (E = +199 mV vs. NHE), and a pure platinum sheet as counter electrode. The exposed active surface area of the working electrode was 5.06 cm^2^, while the electrolyte volume was maintained at 200 mL for all experiments. Electrochemical measurements include: open circuit potential (OCP) monitoring for 1 h, with a measuring period of 2s and general corrosion Rp and Vcorr; scan rate—1 mV/s; overvoltage—40 mV; OCP duration—1 min; determined measure—30 Rp; initial scan—cathodic, allowing the assessment of time-dependent corrosion behavior, passive film stability, and susceptibility to localized corrosion of the alloy in simulated oral environments. Each group was tested a minimum of three times to verify the reproducibility of the measurements. The collected data were subsequently processed and analyzed using Origin 2022 software.

## 3. Results

### 3.1. Surface Morphology and Elemental Composition (SEM–EDX)

The surface morphology and elemental composition of the Zr–2.5Nb alloy before and after 28 days of immersion in artificial saliva with different pH values were investigated by SEM coupled with EDX analysis.

**Figure 1:**
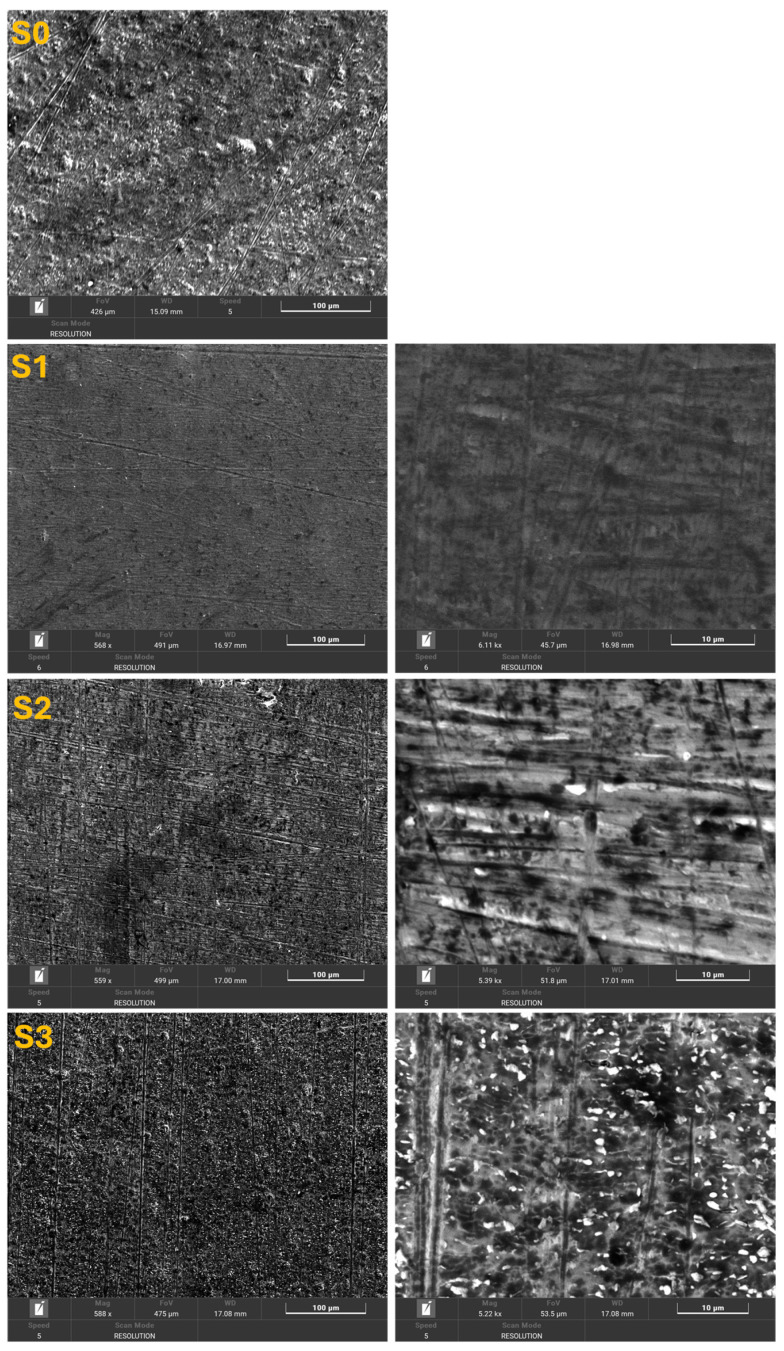
SEM images of Zr-2.5Nb initial (**S0**) and after 28 days immersion in AS with acidic pH (**S1**), neutral pH (**S2**) and alkaline pH (**S3**).

The SEM micrographs illustrate the surface morphology of the Zr–2.5Nb alloy in the initial state (S0) and after 28 days of immersion in artificial saliva with acidic (S1), neutral (S2), and alkaline (S3) pH, as can be seen in Figure 1. Sample S0 (initial condition) presents a relatively homogeneous surface characterized by polishing marks and fine grooves originating from mechanical preparation. The surface appears compact, with no evidence of corrosion products or surface degradation, indicating a stable and passive initial oxide layer. After immersion in acidic artificial saliva (S1), the surface maintains a relatively smooth and uniform appearance at low magnification; however, higher magnification images reveal slight surface etching and attenuation of the polishing lines. The absence of deep pits or cracks suggests that corrosion processes are limited, although localized surface modifications indicate a partial destabilization of the passive film under acidic conditions. In contrast, immersion in neutral artificial saliva (S2) leads to more pronounced surface heterogeneity. SEM images show the persistence of grinding marks combined with the appearance of localized surface deposits and shallow irregularities.

These features suggest the formation of a stable and adherent oxide layer, possibly enhanced by the adsorption of organic components from the artificial saliva, contributing to surface passivation and biomimetic adaptation. The most distinct morphological changes are observed after exposure to alkaline artificial saliva (S3). The surface exhibits increased roughness and a heterogeneous distribution of fine, bright features dispersed across the matrix, indicative of surface precipitation or thickening of the oxide layer. The porous and granular morphology observed at higher magnification is consistent with the formation of corrosion products or bio-inspired mineralized layers, which may enhance surface stability and corrosion resistance in alkaline environments. Overall, SEM analysis demonstrates a clear pH-dependent surface response of the Zr–2.5Nb alloy. While acidic conditions induce minor surface alterations, neutral and alkaline environments promote the development of more complex and protective surface morphologies. These observations support the biomimetic capacity of Zr–2.5Nb to adapt its surface characteristics in response to the chemical conditions of the simulated oral environment, reinforcing its suitability for dental and oral biomedical applications.

**Figure 2:**
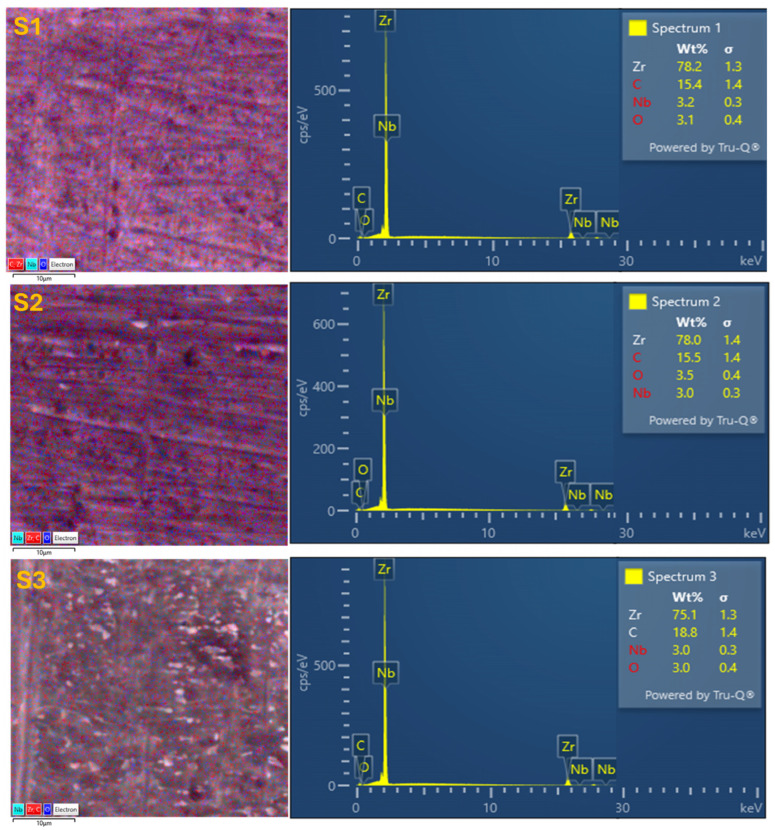
EDX mapping and spectra of Zr-2.5Nb samples after 28 days immersion in AS with acidic pH (**S1**), neutral pH (**S2**) and alkaline pH (**S3**).

The EDX elemental mapping and quantitative spectra obtained after 28 days of immersion in artificial saliva with different pH values provide insight into the chemical stability and surface adaptation of the Zr–2.5Nb alloy, as shown in Figure 2. For the sample immersed in acidic artificial saliva (S1), the EDX spectrum is dominated by zirconium (≈78.2 wt%) and niobium (≈3.2 wt%), confirming the preservation of the alloy’s bulk composition at the surface level. The presence of oxygen (≈3.1 wt%) indicates the formation or persistence of a thin zirconium- and niobium-rich oxide layer, while the detected carbon content (≈15.4 wt%) is attributed to the adsorption of organic species from the artificial saliva and/or residual surface contamination. The relatively low oxygen content suggests a limited oxide thickening under acidic conditions, consistent with the mild surface etching observed in SEM images. In neutral artificial saliva (S2), the elemental composition remains highly stable, with zirconium (≈78.0 wt%) and niobium (≈3.0 wt%) showing minimal variation compared to S1. A slight increase in oxygen content (≈3.5 wt%) indicates enhanced oxide layer stability or gradual oxide growth under near-physiological conditions. The homogeneous distribution of Zr and Nb in the elemental maps supports the formation of a uniform passive film, while carbon (≈15.5 wt%) reflects sustained interaction with organic components of the saliva, contributing to biomimetic surface conditioning. The sample exposed to alkaline artificial saliva (S3) exhibits the most pronounced surface chemical changes. Although zirconium remains the dominant element (≈75.1 wt%), a noticeable increase in carbon content (≈18.8 wt%) is observed, accompanied by stable levels of niobium (≈3.0 wt%) and oxygen (≈3.0 wt%). The elevated carbon signal suggests intensified adsorption or deposition of organic species and possible carbonate-containing compounds under alkaline conditions, in agreement with the granular and heterogeneous surface morphology observed in SEM. These features indicate the development of a more complex surface layer, combining metal oxides and biomimetic organic or inorganic deposits. Overall, EDX analysis confirms the chemical stability of the Zr–2.5Nb alloy across all pH conditions, with no evidence of deleterious elemental leaching. The subtle pH-dependent variations in oxygen and carbon content reflect adaptive surface processes rather than corrosive degradation. These findings support the formation of a protective and biomimetically conditioned surface layer, particularly under neutral and alkaline conditions, which is beneficial for long-term performance in oral and dental biomedical environments.

### 3.2. Surface Chemical Evolution and Biomimetic Interactions (FTIR)

**Figure 3:**
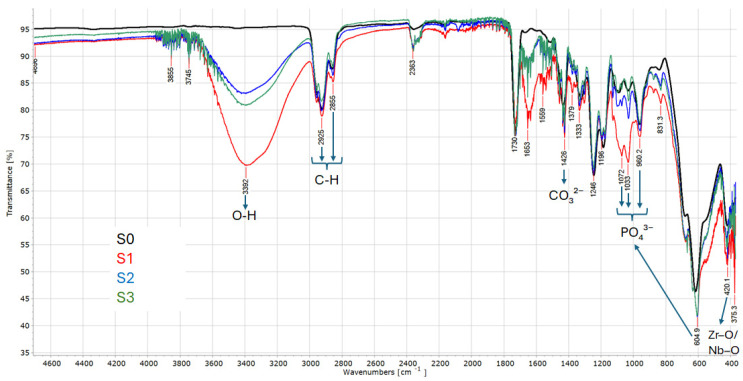
FTIR spectrum of Zr-2.5Nb initial (S0) and after 28 days immersion in AS with acidic pH (S1), neutral pH (S2) and alkaline pH (S3).

FTIR spectra recorded for the Zr–2.5Nb alloy in the initial condition (S0) and after 28 days of immersion in artificial saliva with acidic (S1), neutral (S2), and alkaline (S3) pH reveal significant pH-dependent changes in surface chemistry, reflecting the alloy’s interaction with the simulated oral environment, as shown in Figure 3.

The initial sample (S0) exhibits a relatively flat baseline with weak absorption bands, indicating a clean metallic surface covered by a thin native oxide layer. Low-intensity bands observed below 800 cm^−1^ are attributed to Zr–O and Nb–O vibrations, characteristic of zirconium- and niobium-based passive oxides. After immersion in acidic artificial saliva (S1), a pronounced broad absorption band appears in the 3600–3000 cm^−1^ region, associated with O–H stretching vibrations of adsorbed water molecules and hydroxyl groups. The increased intensity of this band suggests enhanced surface hydration and partial destabilization of the passive oxide layer under acidic conditions. Additionally, bands in the range of 1650–1550 cm^−1^ can be associated with bending vibrations of molecular water and possible weakly adsorbed organic species from saliva. These findings are consistent with SEM observations indicating mild surface etching and limited oxide modification. In neutral artificial saliva (S2), the FTIR spectrum shows a more balanced distribution of absorption bands. The O–H stretching region remains present but with reduced intensity compared to S1, suggesting a more stable hydration state. Bands in the 1400–1000 cm^−1^ range become more distinct and can be attributed to carbonate (CO_3_^2−^) and phosphate-related vibrations, indicating the adsorption or incorporation of saliva-derived inorganic species. This behavior supports the formation of a stable, biomimetically conditioned surface layer under near-physiological conditions. The most complex spectral features are observed for the sample immersed in alkaline artificial saliva (S3). A moderate O–H stretching band is accompanied by intensified peaks in the 1450–1100 cm^−1^ region, which are characteristic of carbonate and phosphate groups, as well as possible C–O stretching vibrations from organic residues. The enhanced intensity and multiplicity of these bands suggest the formation of a thicker and chemically diverse surface layer, combining metal oxides with adsorbed inorganic and organic compounds. This observation correlates well with SEM and EDX results, which revealed increased surface roughness and higher carbon content under alkaline conditions. Overall, FTIR analysis confirms that the surface chemistry of the Zr–2.5Nb alloy evolves dynamically in response to saliva pH. Acidic conditions promote surface hydration and partial oxide destabilization, while neutral and alkaline environments favor the development of chemically complex and stable surface layers enriched with hydroxyl, carbonate, and organic functional groups. These findings highlight the biomimetic surface adaptation of Zr–2.5Nb in simulated oral environments, reinforcing its potential for long-term dental and oral biomedical applications.

Importantly, the enrichment of carbonate and phosphate groups observed by FTIR in neutral and alkaline media provides a direct explanation for the electrochemical behavior of the alloy. The adsorption and partial incorporation of these species into the oxide layer contribute to the stabilization of the surface potential, leading to more noble OCP values. However, the formation of a thicker, chemically heterogeneous layer may also introduce local defects or increased ionic pathways, which can influence charge transfer kinetics and explain the differences observed between OCP and Rp/Vcorr values, particularly under alkaline conditions.

The apparent discrepancy between surface characterization and electrochemical behavior under alkaline conditions can be rationalized by considering the structural and chemical nature of the surface layer formed in S3. SEM–EDX and FTIR analyses indicate the formation of a chemically complex surface layer enriched in oxide, carbonate, and phosphate-related species, accompanied by increased surface roughness and localized precipitates. While such features may initially suggest surface coverage and stabilization, they do not necessarily correspond to the formation of a dense and protective passive film.

Under alkaline conditions, dissolution–reprecipitation processes and enhanced ionic mobility may lead to the growth of thick but porous or weakly adherent surface layers, allowing electrolyte penetration and sustained electrochemical activity at the metal–solution interface. The presence of carbonate- and phosphate-containing deposits may further increase surface heterogeneity, promoting micro-galvanic coupling and under-deposit corrosion phenomena. Consequently, despite the apparent surface enrichment observed by SEM–EDX and FTIR, the electrochemical response reveals progressive degradation, reflected by the pronounced decrease in polarization resistance and the sharp increase in corrosion rate after prolonged exposure.

These findings indicate that, from a biomimetic perspective, surface adaptation in alkaline saliva does not equate to long-term protection, highlighting the distinction between chemical surface modification and effective electrochemical passivation.

### 3.3. Evolution of Artificial Saliva pH During Immersion

The pH evolution of artificial saliva during the immersion of Zr–2.5Nb samples is summarized in Table 1. For acidic artificial saliva (S1), the initial pH of 6.83 showed a gradual increase over time, reaching 7.11 after 14 days and rising markedly to 8.16 after 28 days. This pronounced alkalization suggests active surface–environment interactions, likely associated with ion exchange processes and the formation of corrosion products or oxide layers that modify the local chemical equilibrium. In the case of neutral artificial saliva (S2), the pH exhibited a slight decrease from 7.75 to approximately 7.31–7.34 during the first two weeks, followed by stabilization around 7.40 at 28 days. This relatively stable behavior indicates a balanced interaction between the alloy surface and the surrounding medium, consistent with the formation of a stable passive layer under near-physiological conditions.

For alkaline artificial saliva (S3), the initial pH of 8.22 decreased rapidly within the first 7 days to approximately 7.42 and remained nearly constant thereafter. This convergence toward neutral pH values suggests buffering effects and surface-driven reactions that counteract the initial alkalinity of the solution. Overall, the results demonstrate a clear pH-regulating effect of the Zr–2.5Nb alloy during immersion, highlighting dynamic material–environment interactions that are strongly dependent on the initial chemical conditions of the artificial saliva.

### 3.4. Electrochemical Behavior in Artificial Saliva

**Figure 4:**
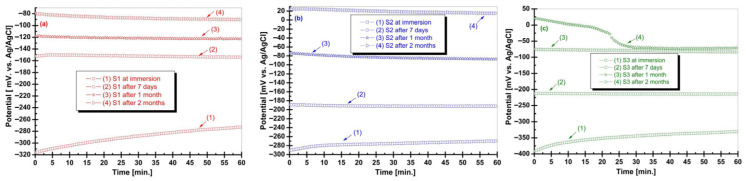
Open circuit potential (OCP) evolution of the Zr–2.5Nb alloy during immersion in artificial saliva with acidic (S1), neutral (S2), and alkaline (S3) pH, recorded at four exposure intervals: initial immersion, 7 days, 1 month, and 2 months. (**a**) Acidic artificial saliva; (**b**) Neutral artificial saliva; (**c**) Alkaline artificial saliva.

The open circuit potential (OCP) evolution of the Zr–2.5Nb alloy was monitored as a function of immersion time in artificial saliva with acidic (S1), neutral (S2), and alkaline (S3) pH at four exposure intervals: initial immersion, 7 days, 1 month, and 2 months can be seen in Figure 4. OCP measurements provide insight into the thermodynamic tendency of the alloy surface toward passivation or active dissolution in simulated oral environments.

In acidic artificial saliva (S1), the initial immersion condition exhibited highly negative OCP values, indicating an electrochemically active surface. With increasing immersion time, a gradual shift toward more positive potential was observed, particularly after 1 month and 2 months of exposure. This trend suggests progressive surface stabilization, likely associated with the formation or reorganization of a passive oxide layer, although the potential remained relatively negative compared to those measured in neutral and alkaline conditions, indicating a higher corrosion susceptibility in acidic media. For samples immersed in neutral artificial saliva (S2), the OCP values were consistently less negative than those recorded in acidic conditions. A clear positive shift in OCP was observed with increasing immersion time, reaching quasi-stable values after prolonged exposure. This behavior reflects the establishment of a stable passive state under near-physiological pH conditions, indicating favorable electrochemical stability of the Zr–2.5Nb alloy in neutral saliva. The most noble OCP values were recorded for samples exposed to alkaline artificial saliva (S3). The initial immersion already showed relatively positive potential, which further increased with immersion time. After 1 and 2 months, the OCP curves displayed a stable plateau, suggesting the formation of a robust and protective passive layer. This behavior indicates enhanced surface stability and reduces thermodynamic driving force for corrosion in alkaline environments. The OCP results demonstrate a pronounced pH- and time-dependent electrochemical response of the Zr–2.5Nb alloy. Acidic conditions delay surface stabilization, whereas neutral and alkaline environments promote faster passivation and more stable electrochemical behavior. These findings support the biomimetic adaptability of the Zr–2.5Nb alloy surface to varying oral pH conditions and provide a foundation for interpreting the subsequent polarization and impedance results.

**Figure 5:**
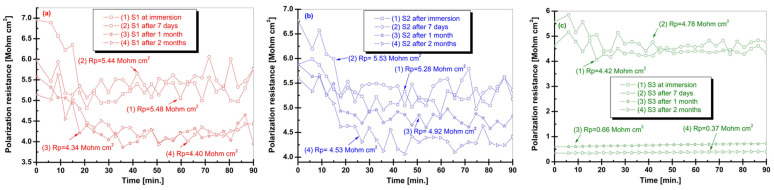
Polarization resistance (Rp) as a function of immersion time for the Zr–2.5Nb alloy in artificial saliva with acidic (S1), neutral (S2), and alkaline (S3) pH, measured at initial immersion, 7 days, 1 month, and 2 months. (**a**) Acidic artificial saliva; (**b**) Neutral artificial saliva; (**c**) Alkaline artificial saliva.

Figure 5 shows the polarization resistance (Rp) evolution of the Zr–2.5Nb alloy monitored as a function of exposure time in artificial saliva with acidic (S1), neutral (S2), and alkaline (S3) pH at four immersion stages: initial immersion, 7 days, 1 month, and 2 months. Rp values provide quantitative information on the corrosion resistance of the alloy, with higher Rp indicating enhanced resistance to electrochemical degradation.

In acidic artificial saliva (S1), the Rp values recorded at initial immersion and after 7 days were relatively high (approximately 5.44–5.48 MΩ·cm^2^), suggesting the presence of a partially protective surface film. However, a noticeable decrease in Rp was observed after prolonged exposure, with values dropping to around 4.34–4.40 MΩ·cm^2^ after 1 and 2 months. This reduction indicates a gradual degradation or destabilization of the passive layer under acidic conditions, consistent with increased corrosion susceptibility observed in other electrochemical measurements. For samples immersed in neutral artificial saliva (S2), Rp values remained comparatively stable throughout the exposure period. Initial and short-term immersion showed Rp values in the range of approximately 5.28–5.53 MΩ·cm^2^, while after 1 and 2 months the resistance slightly decreased but remained relatively high (around 4.92–4.53 MΩ·cm^2^). This behavior reflects the formation and maintenance of a stable passive film under near-physiological pH conditions, indicating favorable corrosion resistance of the Zr–2.5Nb alloy in neutral saliva. In contrast, the alkaline artificial saliva (S3) condition exhibited a distinct Rp response. High Rp values were measured at initial immersion and after 7 days (approximately 4.42–4.78 MΩ·cm^2^), indicating good initial surface protection. However, a pronounced decrease in Rp was observed after prolonged exposure, with values dropping significantly to approximately 0.66 MΩ·cm^2^ after 1 month and further to about 0.37 MΩ·cm^2^ after 2 months. This sharp reduction suggests substantial changes in the surface layer, potentially associated with localized breakdown or increased porosity of the passive film in alkaline environments over extended immersion times. Overall, the Rp results reveal a strong pH- and time-dependent corrosion behavior of the Zr–2.5Nb alloy. Acidic conditions progressively reduce corrosion resistance, neutral conditions favor long-term passive stability, while alkaline environments, despite initially high resistance, may lead to significant degradation of protective surface properties during prolonged exposure. These findings complement the OCP results and highlight the importance of saliva pH in governing the electrochemical performance of Zr–2.5Nb for dental and oral biomedical applications.

**Figure 6:**
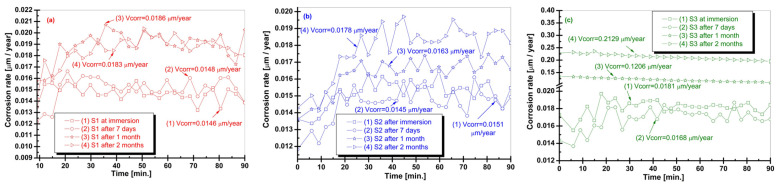
Corrosion rate (Vcorr) evolution of the Zr–2.5Nb alloy determined from electrochemical measurements during exposure to artificial saliva with acidic (S1), neutral (S2), and alkaline (S3) pH at different immersion durations. (**a**) Acidic artificial saliva; (**b**) Neutral artificial saliva; (**c**) Alkaline artificial saliva.

The corrosion rate (Vcorr) of the Zr–2.5Nb alloy was determined from electrochemical measurements as a function of exposure time in artificial saliva with acidic (S1), neutral (S2), and alkaline (S3) pH at four immersion stages: initial immersion, 7 days, 1 month, and 2 months, as can be seen in Figure 6. Vcorr values provide a quantitative assessment of material degradation kinetics, with lower values indicating enhanced corrosion resistance.

In acidic artificial saliva (S1), the corrosion rate exhibited relatively low values at initial immersion and after 7 days (approximately 0.0146–0.0148 μm/year), indicating a temporary protective effect of the surface oxide layer. However, after prolonged exposure, an increase in Vcorr was observed, reaching approximately 0.0186 μm/year after 1 month and remaining at a similar level (0.0183 μm/year) after 2 months. This trend suggests progressive destabilization of the passive layer under acidic conditions, consistent with the decrease in polarization resistance and the increased susceptibility to localized corrosion. For samples immersed in neutral artificial saliva (S2), the corrosion rate remained consistently low and stable throughout the immersion period. Vcorr values ranged between approximately 0.0145 and 0.0178 μm/year, with only minor fluctuations as a function of time. Even after 2 months of exposure, the corrosion rate remained below 0.018 μm/year, confirming the ability of the Zr–2.5Nb alloy to maintain a stable passive state under near-physiological pH conditions. In contrast, the alkaline artificial saliva (S3) condition resulted in a markedly different corrosion behavior. While initial and short-term immersion showed low corrosion rates (0.0168–0.0181 μm/year), a substantial increase in Vcorr was observed after prolonged exposure. After 1 month, the corrosion rate increased to approximately 0.1206 μm/year and further rose to about 0.2129 μm/year after 2 months. This pronounced increase indicates significant degradation of the protective surface layer in alkaline environments over extended immersion times, in agreement with the sharp decrease in polarization resistance observed for S3. The Vcorr results reveal a strong pH- and time-dependent corrosion response of the Zr–2.5Nb alloy. Neutral artificial saliva provides the most favorable conditions for long-term corrosion resistance, while acidic environments induce moderate degradation and alkaline conditions lead to accelerated corrosion during prolonged exposure. These findings complement the OCP and Rp analyses and highlight the critical role of saliva pH in governing the long-term electrochemical performance of Zr–2.5Nb alloy for dental and oral biomedical applications.

Similar pH-dependent trends have been reported for zirconium-based biomaterials exposed to simulated physiological and oral environments. Recent studies have shown that Zr–Nb alloys exhibit enhanced initial passivation in neutral and mildly alkaline media due to surface oxidation and adsorption of carbonate- and phosphate-containing species, while prolonged exposure may lead to chemically complex but structurally heterogeneous surface layers with limited protective efficiency [15,19,21]. In agreement with these reports, the present results indicate that alkaline conditions promote surface enrichment in carbonate and phosphate species, as evidenced by FTIR and SEM–EDX analyses, yet this does not necessarily translate into sustained electrochemical protection. Similar discrepancies between surface chemical enrichment and long-term corrosion resistance have been attributed to porous or weakly adherent deposition layers, dissolution–reprecipitation phenomena, and increased surface heterogeneity, which may facilitate localized corrosion processes [14,16]. These findings support the interpretation that biomimetic surface adaptation under alkaline conditions may involve the formation of thick but defective layers rather than compact, highly protective passive films.

The combined interpretation of SEM/EDX, FTIR, and electrochemical results provides a coherent picture of the pH-dependent biomimetic response of the Zr–2.5Nb alloy. SEM observations revealed distinct surface morphologies as a function of salivary pH, ranging from relatively smooth and compact surfaces under neutral conditions to heterogeneous, deposit-covered morphologies in alkaline media. EDX analysis confirmed surface enrichment in oxygen and minor incorporation of electrolyte-derived species, supporting the formation of chemically modified surface layers. These morphological and elemental changes are consistent with FTIR results, which evidenced the presence of hydroxylated oxide layers as well as carbonate- and phosphate-containing species, particularly under neutral and alkaline conditions. However, electrochemical measures demonstrated that while such surface modifications initially favor passivation, their long-term protective efficiency strongly depends on layer compactness and stability. In alkaline saliva, the formation of chemically complex but porous or weakly adherent layers leads to a progressive decrease in polarization resistance and an increase in corrosion rate, explaining the apparent divergence between surface chemistry and electrochemical performance.

This multi-technique correlation highlights that biomimetic surface adaptation does not necessarily imply sustained electrochemical protection, emphasizing the importance of integrating surface characterization with time-resolved electrochemical analysis when assessing dental biomaterials.

Despite the advantages of artificial saliva models in terms of reproducibility and chemical control, several limitations must be acknowledged when extrapolating the present findings to clinical conditions. Artificial saliva does not fully reproduce the biological complexity of the oral environment, where biofilms, enzymes, proteins, and continuous microbial activity play a critical role in surface chemistry, corrosion mechanisms, and material degradation. Moreover, in vivo conditions are characterized by cyclic pH fluctuations associated with dietary intake, salivary flow, and pathological states, which cannot be fully captured by static immersion protocols.

From a clinical and biomimetic perspective, these factors may significantly influence the stability and functionality of surface layers formed on dental biomaterials. Therefore, future studies should integrate tribocorrosion testing to simulate the combined effects of mechanical wear and electrochemical degradation, as well as biofilm–material interaction studies to assess microbial-mediated corrosion processes. Additionally, exposure protocols involving cyclic pH variations would provide a more realistic simulation of oral conditions and allow evaluation of the durability of surface adaptations observed in the present work.

## 4. Conclusions

This study investigated the biomimetic behavior and corrosion performance of a Zr–2.5Nb alloy during long-term immersion in artificial saliva with acidic, neutral, and alkaline pH, simulating diverse physiological and pathological oral environments. The combined surface and electrochemical analyses demonstrated that saliva pH plays a critical role in governing surface adaptation mechanisms and long-term material stability.

SEM–EDX results revealed a clear pH-dependent surface response. Acidic conditions induced localized surface modifications and partial destabilization of the passive layer, while neutral and alkaline environments promoted the formation of chemically stable surface layers enriched in zirconium- and niobium-based oxides, accompanied by adsorbed organic and inorganic species. FTIR analysis confirmed the evolution of surface chemistry through the presence of hydroxyl, carbonate, and organic-related functional groups, highlighting the ability of the alloy to undergo biomimetic surface conditioning in response to the surrounding environment.

Electrochemical measurements further supported these observations. Open circuit potential monitoring indicated progressive surface stabilization over time, particularly in neutral and alkaline saliva. Polarization resistance and corrosion rate analyses demonstrated that neutral saliva provided the most favorable conditions for long-term corrosion resistance, maintaining high Rp values and low corrosion rates throughout the immersion period. Acidic saliva led to moderate degradation over time, while alkaline artificial saliva initially promoted surface stabilization, prolonged exposure resulted in a pronounced decrease in polarization resistance and a significant increase in corrosion rate, indicating that the surface layer formed under alkaline conditions is likely porous or weakly adherent rather than a dense passive film. In contrast, neutral saliva provided the most stable long-term electrochemical behavior, confirming its role in sustaining protective passivation of the Zr–2.5Nb alloy. Overall, the results confirm that the Zr–2.5Nb alloy exhibits strong biomimetic adaptability and good corrosion resistance under neutral and mildly aggressive oral conditions, supporting its potential suitability for dental and oral biomedical applications. However, the findings also underline the importance of considering pH fluctuations in the oral environment, particularly under prolonged alkaline conditions, when evaluating the long-term performance of zirconium-based biomaterials. This work contributes to a deeper understanding of pH-driven surface adaptation mechanisms and provides valuable guidance for material selection and surface optimization strategies in dental biomaterial design.

Future research should focus on tribocorrosion and biofilm-related studies, as well as long-term exposure under cyclic pH conditions, to better replicate the complexity of the oral environment. Additionally, surface engineering strategies and biological evaluations are needed to further optimize the corrosion resistance and clinical performance of Zr–2.5Nb alloys for dental applications.

## Figures and Tables

**Table 1:** pH values of artificial saliva during the immersion of Zr–2.5Nb samples.

Sample	pH Value			
	1 day	7 days	14 days	28 days
S1 Acidic artificial saliva	6.83	6.85	7.11	8.16
S2 Neutral artificial saliva	7.75	7.34	7.31	7.40
S3 Alkaline artificial saliva	8.22	7.42	7.41	7.40

## Data Availability

All data analyzed during this study are included in this published article and its.
